# Treatment of rickets and dyslipidemia in twins with progressive familial intrahepatic cholestasis type 2

**DOI:** 10.1186/s13633-020-00079-1

**Published:** 2020-05-26

**Authors:** Sunitha R. Sura, Emily L. Germain-Lee

**Affiliations:** 1 Connecticut Children’s Medical Center, 505 Farmington Avenue, Farmington, CT 06032 USA; 2 Department of Pediatrics, University of Connecticut School of Medicine, 505 Farmington Avenue, Farmington, CT 06032 USA

**Keywords:** PFIC2, Rickets, Dyslipidemia

## Abstract

**Background:**

Progressive Familial Intrahepatic Cholestasis Type 2 (PFIC2) is a rare congenital cholestatic liver disease that progresses to end stage liver disease. It is associated with fat soluble vitamin D deficiency rickets and severe dyslipidemia; however, treatment of these secondary effects remains a challenge.

**Case presentation:**

One year old twin males born to a mother with intrahepatic cholestasis during pregnancy presented with jaundice, pruritus and failure to thrive. Lab evaluation revealed significant transaminitis, direct hyperbilirubinemia and normal gamma glutamyl transferase (GGT). Genetic studies confirmed PFIC2. Further evaluation for fat soluble vitamin deficiencies revealed severe vitamin D deficiency rickets. High dose vitamin D replacement therapy using Ergocalciferol (Vitamin D_2_) 50,000 IU three times a week over 10 weeks led to the improvement of Vitamin D, 25-Hydroxy (25-OH) serum levels and resolution of rickets. Dyslipidemia with very low high density lipoprotein-cholesterol (HDL-C) and high triglycerides was more profound in our patients compared to what has been described in the literature thus far. The dyslipidemia improved 2 months after internal biliary diversion.

**Conclusions:**

Higher doses of Vitamin D therapy are needed for treatment of rickets secondary to cholestasis. Extremely low HDL-C levels are characteristic of PFIC and improve with treatment of underlying cholestasis. Maternal intrahepatic cholestasis during pregnancy can be an early warning sign.

## Background

PFIC is a rare autosomal recessive group of disorders associated with different genetic etiologies that occurs due to the inability to form and excrete bile from hepatocytes. This congenital cholestatic liver disease has an incidence of about 1 per 50,000 to 100,000 births [1]. PFIC1 is caused by a mutation in *ATP8B1,* which encodes aminophospholipid transporting ATPase. PFIC2 is caused by a mutation in *ABCB11*, the gene that encodes the bile salt export pump (BSEP). Deficiency in BSEP is the most common form of PFIC that presents in early life with jaundice, high serum bile acids and transaminases, normal GGT, pruritis, and failure to thrive. PFIC2 typically progresses to end stage liver disease and has up to a 15% risk of hepatocellular carcinoma by 5 years of age [2]. Non progressive forms include benign recurrent intrahepatic cholestasis, intrahepatic cholestasis of pregnancy and drug induced cholestasis. PFIC genes are associated with intrahepatic cholestasis of pregnancy in families [3]. Cholestasis secondary to PFIC is accompanied by fat-soluble vitamin deficiency that can lead to Vitamin D, 25-OH deficiency rickets [4] and lipid disturbances. This dyslipidemia specifically includes elevated triglycerides and low HDL-C with an increased risk of atherosclerosis [5].

## Case presentation

We present monochorionic-diamniotic twin males of Pakistani origin born to consanguineous parents who presented with jaundice, pruritus, and failure to thrive at 1 year of age. They were induced at 34 weeks gestation secondary to maternal intrahepatic cholestasis of unknown etiology during pregnancy. Mother was on ursodeoxycholic acid since 13 weeks gestation. Work up in the twins revealed significant transaminitis. Twin A had elevated levels of the following: Aspartate Transaminase (AST) =1670 U/L (10–55), Alanine Transferase (ALT) = 967 U/L (10–55), total bilirubin = 5.5 mg/dL (0.2–1.0), direct bilirubin = 3.7 mg/dL (0–0.2), prolonged coagulation with prothrombin time (PT) =15.2 s (9.5–13) and partial thromboplastin time (PTT) = 31 s (25–36), total serum bile acids =245 μmol/L (0–19), and a normal GGT 26 U/L (11–50). There was also hepatosplenomegaly. Twin B had elevated levels of the following: AST =1117 U/L, ALT = 689 U/L, total bilirubin = 2.8 mg/dL, direct bilirubin =1.5 mg/dL, PT =13.8 s, PTT = 46 s, total serum bile acids = 125 μmol/ and a normal GGT 18 U/L. Lipid profile revealed a low HDL-C and high triglycerides. Urine analysis using fast atom bombardment ionization mass spectrometry (FAB-MS) showed no defect in the cholesterol-bile acid biosynthetic pathway. Treatment with oral ursodeoxycholic acid 100 mg twice daily was initiated.

The genetic cholestasis panel revealed homozygous mutations of *ABCB11*, specifically c.3803G > A (p.R1268Q) confirming PFIC2 [6]. Further work-up demonstrated fat soluble vitamin deficiencies including Vitamin D, 25-OH deficiency with radiologic findings of severe rickets. AquADEKs 2 mL daily and Vitamin K 15 mg daily were started. Vitamins A and E as well as PT, PTT and international normalized ratio (INR) were monitored and normalized with treatment. Both twins had similar clinical features and biochemical lab results. Physical examination revealed fussy infants with icteric sclera, multiple scratch marks on skin, widely open anterior fontanelles, widening of the wrists, and hepatomegaly with the liver edges palpable 4 cm below the right costal margin in both twins. Abdominal ultrasound confirmed hepatomegaly with no intra or extrahepatic biliary dilation or cholelithiasis in both twins.

**Fig. 1:**
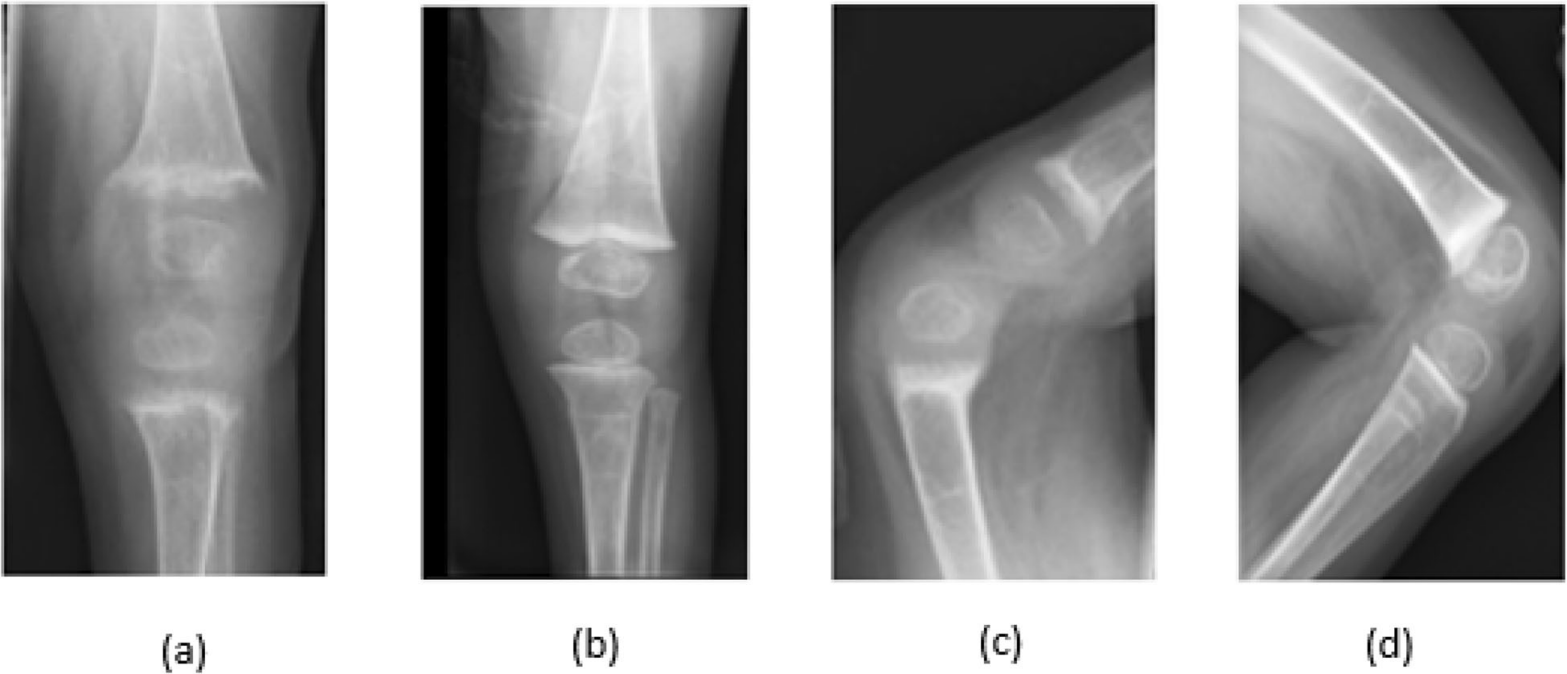
*Twin* A knee radiographs. AP view (**a**) before treatment (**b**) after treatment. Lateral view (**c**) before treatment and (**d**) after treatment. **a** and **c** show diffuse osteopenia, fraying and widening of metaphyses. **b** and **d** demonstrate improvement in rickets

**Fig. 2:**
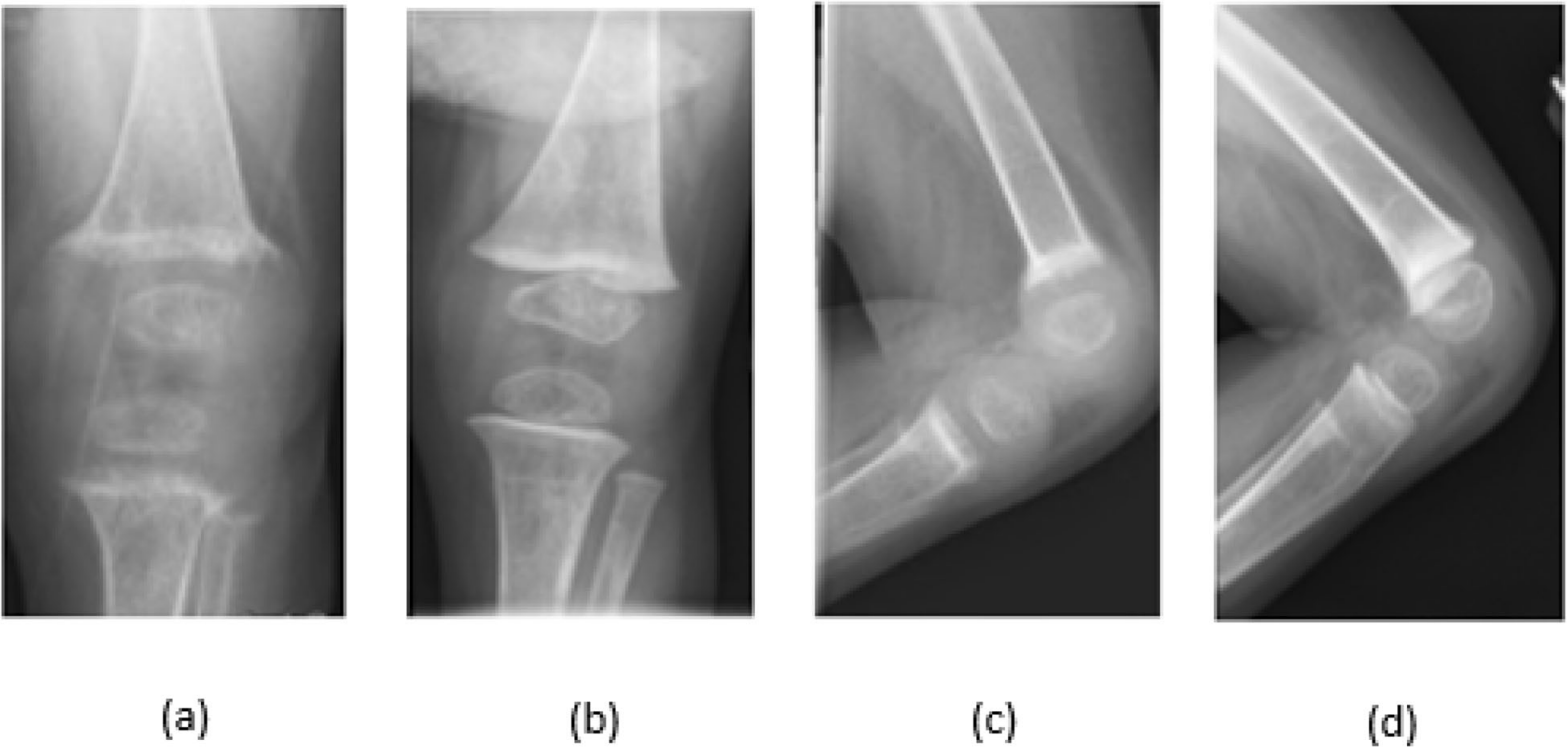
*Twin* B knee radiographs. AP view (**a**) before treatment (**b**) after treatment. Lateral view (**c**) before treatment and (**d**) after treatment. **a** and **c** show diffuse osteopenia, fraying and widening of metaphyses. **b** and **d** demonstrate improvement in rickets

Knee radiographs showed diffuse osteopenia, marked fraying and widening of the distal femoral and proximal tibial/fibular metaphyses as shown in Fig. 1a, c, Fig. 2a and c. The Vitamin D, 25-OH levels in Twin A and Twin B were low at 17.5 nmol/L and 15 nmol/L respectively. They both had elevated parathyroid hormone (PTH) levels of 47.3 pmol/L and 36.6 pmol/L respectively, low phosphorus, and appropriately elevated Vitamin D, 1,25-Dihydroxy levels of 235.2 pmol/L and 256.8 pmol/L (98 pg/mL and 107 pg/mL respectively). Alkaline phosphatase levels were elevated at 1137 and 1640 U/L respectively. Bone specific alkaline phosphatase levels were elevated at 241.4 μg/L and 405 μg/L respectively (reference range 25.4–124 μg/L). Vitamin D_2_ 50,000 IU weekly was initiated. Both twins developed hypocalcemia after initiation of Vitamin D supplements. Serum total calcium levels decreased from 2.18 mmol/L to 1.95 mmol/L and 2.08 mmol/L to 1.65 mmol/L respectively. Activated Vitamin D (calcitriol) was initiated for hypocalcemia and then discontinued when serum total calcium levels and PTH levels were improving and urinary calcium excretion was noted to trend up, although still within the normal range. Details of the treatment regimen are shown in Table 1.

**Table 1:** Metabolic bone labs and treatment regimens in Twin A and B (Conventional unit values are represented in parentheses)

Time	Twin	Vitamin D, 25-Hydroxy: 75–250 nmol/L (30–100 ng/mL)	Total Calcium, serum: 2.13–2.65 mmol/L (8.5–10.6 mg/dL)	Phosphorus: 1.45–2.16 Mmol/L (4.5–6.7 mg/dL)	PTH, intact 1.6–6.9 pmol/L (15–65 pg/mL)	Alkaline Phosphatase 104–345 U/L	Treatment comments
Day 0	A	17.5 (7)	2.18 (8.7)	0.77 (2.4)	47.3 (446)	964	Started Ergocalciferol 50,000 IU weekly and Calcium carbonate (Ca carb) elemental Ca at 50 mg/kg/day
	B	15 (6)	2.08 (8.3)	0.74 (2.3)	36.6 (345)	1367	
Day 5	A		1.95 (7.8)	0.90 (2.8)	40.4 (381)	599	Started Calcitriol 0.2 mcg bid due to drop in serum total calcium levels and elevated PTH levels. Increased Ca carb to 100 mg/kg/day. Started sodium phosphate 2 mmol.kg/day.
	B		1.65 (6.6)	0.94 (2.9)	51.8 (488)	923	
Day 7	A		2.10 (8.4)	0.94 (2.9)	46.3 (436)	610	Increased Calcitriol dose to 0.4 mcg bid due to persistent hypocalcemia (Twin A ionized Ca 0.99 and Twin B iCa 0.92. (normal 1.14–1.40 mmol/L), further increase in PTH levels. Urine Ca/Creatinine ratio: Twin A- 0.08; Twin B- 0.1 (normal 0.6)
	B		1.78 (7.1)	1.10 (3.4)	65.6 (618)	1014	
Day 10	A		2.2 (8.8)	0.77 (2.4)	39.5 (372)	692	Decreased Calcitriol to 0.4 mcg qd and discontinued Sodium phosphate. (serum phos trending down due to additional phos possibly stimulating FGF23 and further exacerbating phosphaturia. Underlying issue for low phos is secondary hyperparathyroidism from Vitamin D deficiency)
	B		1.93 (7.7)	0.87 (2.7)	48.8 (460)	1113	
Week 3	A		2.38 (9.5)	0.68 (2.1)	33.1 (312)	878	Discontinued Calcitriol as serum total calcium levels and PTH were improving. Increased Ergocalciferol to 50,000 IU two times/week as PTH still elevated after 3 doses of Vitamin D_**2**_. Decreased Calcium carb to 50 mg/kg/day. Discharged to home.
	B		2.33 (9.3)	0.84 (2.6)	36.2 (341)	1759	
Week 5	A	15 (6)	2.50 (10)	0.61 (1.9)	12.3 (116)	755	Twins were readmitted for G tube placement and liver biopsy. They missed Ergocalciferol doses at home. Increased Ergocalciferol to 50,000 IU three times/week Restarted Calcitriol at 0.1 mcg bid. Discharged after 1 week.
	B	< 12.5 (< 5)	2.35 (9.4)	0.58 (1.8)	26.8 (253)	1367	
Week 10	A	22.5 (9)	2.68 (10.7)	1.74 (5.4)	6.6 (62)	743	Twins were seen outpatient and decreased Calcitriol to 0.1 mcg qd. Family adherent to Vitamin D therapy. Week 12: Internal biliary diversion surgery was performed.
	B	25 (10)	2.53 (10.1)	1.39 (4.3)	10.5 (99)	849	
Week 14	A	32.5 (13)	2.70 (10.8)	1.91 (5.9)	1.4 (13)	341	Discontinued Calcitriol and decreased Ca to 30 mg/kg/day. Continued Ergocalciferol 50,000 IU three times/week. Urine Ca/Creat: Twin A- 0.21; Twin B 0.26
	B	40 (16)	2.85 (11.4)	1.81 (5.6)	2.0 (19)	333	
Week 15	A		2.55 (10.2)	1.74 (5.4)	2.9 (27)	287	Switched to Ergocalciferol 16,000 IU daily and continued Ca carb at 30 mg/kg/day for 2 weeks following which they were switched to maintenance dose of Vitamin D_3_ 2000 IU daily and 25-OH Vitamin D levels were monitored.
	B		2.53 (10.1)	1.94 (6)	3.6 (34)	302	

The Vitamin D, 25-OH levels for Twin A and Twin B continued to be low at 15 nmol/L and < 12.5 nmol/L respectively despite weekly ergocalciferol 50,000 IU for 4 weeks. Ergocalciferol was increased to 50,000 IU three times a week for 10 weeks which resulted in improvement in Vitamin D, 25-OH levels to 32.5 and 40 nmol/L respectively. Other parameters including calcium, phosphorus, alkaline phosphatase, and PTH improved as shown in Table 1. Radiologic findings of rickets improved as shown in Fig. 1b, d, Fig. 2b and d. Liver biopsy in both twins showed hepatocellular and canalicular cholestasis with bile plugs, patchy hepatocellular swelling, mild portal/lobular inflammation and portal/periportal/sinusoidal fibrosis. Electron microscopy of liver specimens showed dilated canaliculi with finely granular bile consistent with PFIC2.

The lipid profiles performed at initial diagnosis were consistent with severe dyslipidemia in the twins with elevated total cholesterols of 5.8 mmol/L and 4.61 mmol/L respectively. They had elevated triglcyeride (TG) levels of 2.9 mmol/L and 3.3 mmol/L. HDL-C levels were extremely low at 0.1 mmol/L and 0.16 mmol/L. Low density lipoprotein-cholesterol (LDL-C) levels were elevated at 4.3 mmol/L and 2.93 mmol/L respectively. They had persistent pruritus and underwent an internal biliary diversion procedure involving a jejunal interposition graft between the gallbladder and splenic flexure of the colon and jejunojejunal anastomosis (cholescystojejunostomy). Pruritus and transaminitis improved after surgery. Labs obtained 1 week after surgery showed Twin A’s AST =159 U/L, ALT =155 U/L, total bilirubin = 3 mg/dL and direct bilirubin =2.3 mg/dL. Twin B had a similar response with reduction in AST =104 U/L, ALT =117 U/L, total bilirubin =2.2 mg/dl and direct bilirubin =1.7 mg/dl. The LDL-C normalized, TG levels and HDL-C levels improved 2 months after surgery as shown in Table 2.

**Table 2:** Lipid abnormalities before and after internal biliary diversion surgery (Conventional unit values are represented in parentheses)

Lipids	Twin A		Twin B	
	Before surgery	Two months after surgery	Before surgery	Two months after surgery
Total cholesterol: < 5.18 mmol/L (< 200 mg/dL)	5.80 (224)	3.60 (139)	4.61 (178)	2.72 (105)
Triglycerides: < 1.7 mmol/L (< 150 mg/dL)	2.9 (254)	1.9 (168)	3.3 (296)	0.8 (69)
HDL-C: > 1.01 mmol/L (> 39 mg/dL)	0.10 (4)	0.26 (10)	0.16 (6)	0.31 (12)
LDL-C: < 3.37 mmol/L (< 130 mg/dL)	4.38 (169)	2.46 (95)	2.93 (113)	2.05 (79)

## Discussion

Vitamin D deficiency is common in cholestasis and treatment remains a challenge compared to children without cholestasis. We initiated therapy with Vitamin D_2_ 50,000 IU weekly and planned to continue it for 6 weeks as per standard guidelines [7, 8]. Although the guideline states that Vitamin D_3_ has a longer half-life when used in bolus doses compared to Vitamin D_2_, we chose to use vitamin D_2_ as it is readily available in our hospital pharmacy at a concentration of 8000 IU/mL and the necessary doses can be provided orally in a much lower volume than that of vitamin D3 for our young patients. Also, vitamin D_2_ has been shown to be effective in increasing serum vitamin D, 25-OH levels similar to vitamin D_3_ [9].

After 4 weeks of therapy, we did not see improvement in Vitamin D, 25-OH levels. In liver disease, vitamin D deficiency results from vitamin D malabsorption in the absence of bile salts in the intestinal lumen and failure of vitamin D 25-hydroxylation by microsomal or mitochondrial liver cells. Treatment of vitamin D deficiency in hepatic rickets requires large doses of vitamin D 10–20 times higher than the RDA. Higher doses can be used as long as the Vitamin D, 25-OH levels are being monitored to avoid toxicity [10, 11]. Children with chronic liver disease secondary to Wilson’s disease, chronic hepatitis B, autoimmune hepatitis, glycogen storage disease and non alcoholic fatty liver disease have also been shown to have increased prevalence of vitamin D deficiency, insufficiency and rickets. In these children, high dose vitamin D_3_ therapy using weekly 60,000 IU for 3 months was shown to be more efficacious than stoss regimen using a single dose of 600,000 IU of vitamin D_3_ and daily maintenance dose of 600 IU daily for 3 months [12]. To our knowledge this is the first report describing use of high dose Ergocalciferol (50,000 IU three times a week) until acceptable levels of Vitamin D, 25 hydroxy are achieved to treat severe rickets in PFIC2. It is key to treat the vitamin D deficiency adequately as osteopenia and bone fractures have been described in children with cholestatic liver disease from PFIC, Alagille syndrome and biliary atresia [13]. We would like to emphasize that high dose Vitamin D supplementation therapy needs to be considered only in the treatment of rickets from cholestasis that does not improve with therapy using standard guidelines and not for routine supplementation in neonatal cholestasis. We noticed marked improvement in metabolic bone parameters between the fifth and tenth week when the vitamin D levels started to improve. We used calcium carbonate and titrated the dose based on the metabolic bone parameters. Calcitriol was used during the initial part of therapy for hypocalcemia and management of rickets. Dose was adjusted based on lab parameters [14]. Spot urine Ca/Creatinine ratio was closely monitored and levels were maintained in the normal range [15].

Families with mutations in *ABCB11,* as in our case, have been shown to have a spectrum of diseases including benign recurrent intrahepatic cholestasis, intrahepatic cholestasis of pregnancy, drug-induced cholestasis, hepatocellular carcinoma, and PFIC2. These conditions are due to partially impaired function of BSEP in heterozygous carriers. Estrogen has been shown to be a trigger for predisposed individuals, and 27 % of women with intrahepatic cholestasis of pregnancy have pruritus with oral contraceptives. Other triggers for cholestasis in heterozygous carriers include pregnancy, infections and drugs. Mutation carriers have an increased risk of cholelithiasis but they have normal labs in between cholestatic attacks [3]. Interestingly, our patients’ mother, who was a heterozygous carrier, presented with intrahepatic cholestasis of pregnancy during both her pregnancies, however she was more symptomatic with the twin pregnancy. In retrospect, these complications were early indications of the underlying genetic etiology in the mother and her twins.

Low Vitamin D,25-OH levels have been associated with elevated triglycerides and low HDL-C levels in obese as well as non-obese children [16]. However, HDL-C is characteristically low in PFIC compared to other cholestatic diseases. This low HDL-C can be secondary to low lecithin-cholesterol acyltransferase (LCAT) activity, decreased hepatic synthesis of apolipoprotein A-1 (Apo-A1) and decreased hepatic lipase activity secondary to the intrahepatic cholestasis [5, 17]. Nagasaka et al. studied 5 patients with PFIC and Janowska et al. examined 26 patients with this condition, and both cohorts had low HDL-C levels with average ranges of 0.52 +/− 0.08 mmol/L (20+/− 3 mg/dl) and 0.74 +/− 0.16 mmol/L (28.57 +/− 6.33 mg/dl) respectively [5, 18]. Our patients, however, had profoundly low levels of HDL-C of 0.1 mmol/L and 0.16 mmol/L which is much lower than what has been described in the literature with PFIC. No correlation between this specific mutation and lipid phenotype has been described before. HDL-C and Apo-A1, a major protein in HDL, play key roles in reverse cholesterol transport and have been shown to reduce risk for atherosclerosis [19]. Patients with PFIC have an increased risk for atherosclerosis as evidenced by increased carotid intimal thickness [5]. Our patients had improvement in all lipid parameters 2 months after internal biliary diversion. LDL-C, a known risk factor for atherosclerosis normalized in both twins after surgery and triglycerides also improved. However, the twins will need to be monitored for the development of atherosclerosis, as will their parents.

## Conclusions

Children with PFIC should be screened for vitamin D deficiency rickets and dyslipidemia. Close follow- up and gradual escalation of vitamin D therapy with monitoring is needed for resolution of rickets. Dyslipidemia improves with improvement of cholestasis. Maternal intrahepatic cholestasis during pregnancy can be an early warning sign.

## Data Availability

Data sharing not applicable to this article as no datasets were generated or analyzed during the current study.

## Abbreviations

PFIC2: Progressive Familial Intrahepatic Cholestasis Type 2
GGT: Gamma Glutamyl Transferase
HDL-C: High Density Lipoprotein- Cholesterol
BSEP: Bile Salt Export Pump
AST: Aspartate Transaminase
ALT: Alanine Transferase
PT: Prothrombin Time
PTT: Partial Thromboplastin Time
FAB-MS: Fast Atom Bombardment ionization- Mass Spectrometry
INR: International Normalized Ratio
PTH: Parathyroid Hormone
Ca: Calcium
Ca carb: Calcium carbonate
FGF-23: Fibroblast Growth Factor-23
LDL-C: Low Density Lipoprotein- Cholesterol
LCAT: Lecithin-Cholesterol Acyl Transferase
Apo-A1: Apolipoprotein A-1
